# Factors related to successful outcome of conservative treatment for rotator cuff tears

**DOI:** 10.3109/03009734.2010.493246

**Published:** 2010-07-19

**Authors:** Minoru Tanaka, Eiji Itoi, Katsumi Sato, Junichiro Hamada, Shin Hitachi, Yuichi Tojo, Masahito Honda, Shiro Tabata

**Affiliations:** ^1^Tohoku Rosai Hospital, SendaiJapan; ^2^Department of Orthopaedic Surgery, Tohoku University School of Medicine, SendaiJapan; ^3^Kuwano Kyoritsu Hospital, KoriyamaJapan; ^4^Department of Diagnostic Radiology, Tohoku University School of Medicine, SendaiJapan; ^5^Takeda General Hospital, AizuwakamatsuJapan; ^6^Iwaki Chuo Hospital, IwakiJapan

**Keywords:** Conservative treatment, operative treatment, prognostic factors, rotator cuff tears

## Abstract

**Background:**

Much controversy exists as to the management of full-thickness tears of the rotator cuff. Not all patients with rotator cuff tears require surgical treatment. We have little information whether there are factors that are related to successful outcome of conservative treatment.

**Aim:**

The purpose of this study was to determine the factors related to the successful outcome following conservative treatment.

**Methods:**

This study included 123 shoulders in 118 patients with full-thickness tears of the rotator cuff diagnosed by high-resolution magnetic resonance imaging with a microscopy coil. All patients were treated conservatively for at least 3 months. Clinical symptoms improved in 65 shoulders in 62 patients by conservative treatment (conservative group), but remained unchanged or aggravated in 58 shoulders in 56 patients, who eventually underwent surgical repair (surgical group).

**Results:**

The following parameters showed significant differences: 1) integrity of the intramuscular tendon of the supraspinatus (24.1% in the surgical group and 58.4% in the conservative group showed an intact intramuscular tendon); 2) supraspinatus muscle atrophy (occupancy ratio was 69.8% in the surgical group and 78.0% in the conservative group); 3) impingement sign (positive in 79.3% in the surgical group and 30.7% in the conservative group); and 4) external rotation angle (35.0 degrees in the surgical group and 52.2 degrees in the conservative group). The success rate of conservative treatment was 87% in the cases with at least three of these four factors.

**Conclusion:**

These four factors are useful in selecting patients who will respond well to conservative treatment before initiating the treatment.

## Introduction

Rotator cuff tears are common shoulder disorders. However, some issues regarding treatment of this disease remain controversial. There are various opinions regarding the treatment intervention for full-thickness tears of the rotator cuff. Numerous studies have demonstrated the benefit of surgical management of this disorder (1–10), whereas some investigators have recommended conservative management as the first choice rather than surgical treatment (11–13). In fact, not all patients with this disease actually require surgical treatment. Older patients in their seventh, eighth, or ninth decade of life with chronic tears and low quality tendons and muscles should be treated conservatively (14).

There have been many reports about the outcome of conservative treatment of symptomatic full-thickness rotator cuff tear (13,15–29). The success rate of conservative treatment varies from 33% to 88% (13,17,20,23–26). This variability is probably the result of differences in the indication for conservative and surgical treatments, because few studies have clearly indicated the inclusion/exclusion criteria of patients for conservative treatment (12,20,23). As a result, there is little information regarding which factors are related to the successful outcome following conservative treatment.

We utilized a microscopy coil to make a diagnosis of rotator cuff tears with magnetic resonance image (MRI). This MR technique, which provides very fine resolution and high-contrast images, enables us to visualize the intramuscular tendon of the rotator cuff. The intramuscular tendon, which most of the muscle fibers attached (30), seems to be an important structure for transmitting force to the greater tuberosity. Therefore, it is likely that the integrity of intramuscular tendon affects the results of conservative treatment for rotator cuff tears. The purpose of this study was to determine the factors related to the successful outcome following conservative treatment for full-thickness tears of the rotator cuff.

## Patients and methods

**Figure 1.**
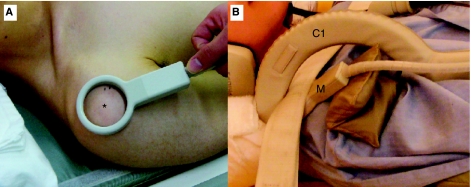
A patient with rotator cuff tear undertaking high-resolution magnetic resonance imaging with a microscopy coil. A: Confirm the intertubercular groove and greater tuberosity. Set the microscopy coil on the greater tuberosity. Asterisk indicates the location of the greater tuberosity. B: At first, the C1 coil (C1) is set on the shoulder and then the microscopy coil (M) is put on the greater tuberosity.

A total of 527 patients with symptomatic shoulder disability were referred to the first author's facility between August 2003 and December 2006. From these patients, 128 patients who were diagnosed with full-thickness tears of the rotator cuff by 1.5-tesla high-resolution MRI with a microscopy coil (Gyroscan Intera Nova 1.5T; Philips Medical Systems, Ienthoven, The Netherlands) were enrolled in this retrospective study (Figure 1). There were 67 males and 61 females, aged from 42 to 83 years (average 69 years) with traumatic or non-traumatic rotator cuff tears. We recorded an onset of shoulder pain, tenderness, painful arc, supraspinatus test, and impingement sign. The diagnosis of rotator cuff tear was confirmed on high-resolution MRI with a microscopy coil. Patients with traumatic glenohumeral dislocation, fracture dislocation, instability, and other significant intra-articular disorders such as synovial chondromatosis associated with rotator cuff tears and with diabetes mellitus and combined tears of subscapularis and/or teres minor tendons were excluded from this study.

All patients were initially treated conservatively for at least 3 months. Conservative treatment included various combinations of rest, non-steroidal anti-inflammatory drugs, and the subacromial injection of steroids with lidocaine. After the acute inflammation of the glenohumeral joint had subsided, physical therapy, such as massage, stretching exercise of rotator cuff and scapular muscle, passive range of motion, and muscle strengthening exercises, was started. The goal of the physical therapy included ensuring achievement of good range of motion at the glenohumeral joint with proper glenohumeral and scapulothoracic kinematics, appropriate scapular stabilization, and improvement of static and dynamic muscular strength stability of the rotator cuff. We used therapeutic modalities such as ultrasound, electrical stimulation, and manual therapy. After at least 3 months of conservative treatment, the same observer (M.T.) performed the clinical evaluations according to the Constant score (31). The total Constant score was defined as excellent (Constant score >85), good (Constant score 75–84), fair (Constant score 65–74), and poor (Constant score <64). Average of duration of conservative treatment was 3.7 months (range, 3–10 months).

Among the patients with improved symptoms, 65 shoulders in 62 patients with follow-up periods longer than 2 years were evaluated by personal interview and physical examination for this study. The other 10 shoulders in 10 patients observed for less than 2 years were excluded from this study. We classified the patients into two groups: the conservative group of 65 shoulders in 62 patients with an excellent or good outcome which was maintained for longer than 2 years, and the surgical group of 58 shoulders in 56 patients who underwent surgical treatment due to unsatisfactory results after conservative treatment. The average follow-up period was 2.4 years (range, 2–3.5 years) for the conservative group and 2.2 years (range 1.2–3.2) for the surgical group.

The two groups were compared regarding the following factors: age, gender, history of trauma, night pain, range of motion in forward elevation, range of motion in external rotation, impingement sign, and MRI findings including torn tendons, tear size, muscle atrophy of the supraspinatus, and integrity of the intramuscular tendon of the supraspinatus.

**Figure 2.**
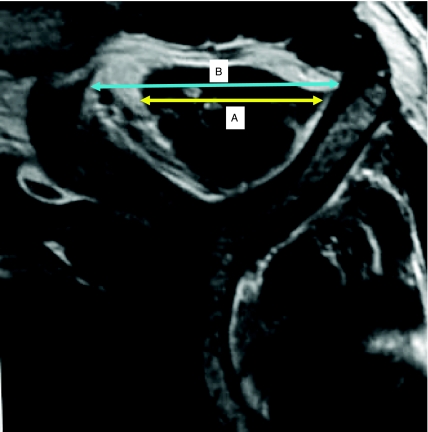
Muscle atrophy measured by the cross-sectional area of the supraspinatus fossa on sagittal oblique MRI. The occupancy ratio of the supraspinatus A/B × 100 (%). A = maximum width of the supraspinatus. B = maximum width of the supraspinatus fossa.

**Figure 3.**
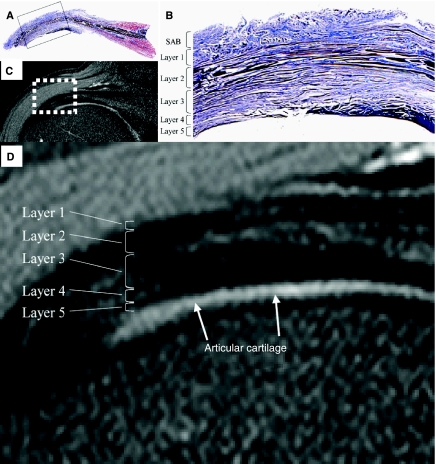
Correlation of five-layer structure of rotator cuff between the histologic specimen and MR images. A, B: Histologic specimen of the supraspinatus tendon (Elastica-Masson stain). SAB = tissue of subacromial bursa. C, D: High-resolution MR images demonstrate the corresponding laminar structure of the supraspinatus tendon (Fat-suppressed short-TE T2-weighted images: TR/TE = 1800/50).

**Figure 4.**
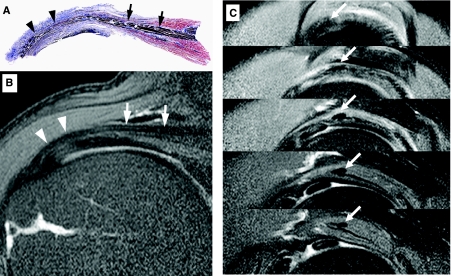
Intramuscular tendon of the supraspinatus. A: The intramuscular tendon, which is in the supraspinatus muscle belly (black arrows), extends to the greater tuberosity. At the tendinous region, the intramuscular tendon is continuous to the layer 2 (black arrowheads). B: This bundle is recognized as low signal intensity band on the MR image with a microscopy coil (white arrows and arrowheads). C: Integrity of the intramuscular tendon of the supraspinatus was evaluated on sagittal oblique image of MRI. The intramuscular tendon is recognized as an elliptic area of low signal intensity (white arrows).

**Figure 5.**
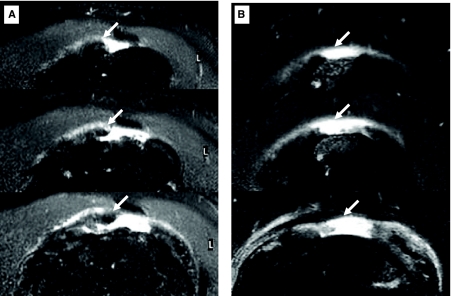
Integrity of the intramuscular tendon of the supraspinatus. A: Intact intramuscular tendon with torn supraspinatus tendon. An elliptic area of low signal intensity of the intramuscular tendon was identified (white arrows). B: Intramuscular tendon tear of a case with torn supraspinatus tendon. An elliptic area of low signal intensity was not identified (white arrowheads).

Passive range of motion was measured in forward elevation and external rotation. Neer's maneuver and Hawkins' maneuver were used to assess impingement sign. The torn tendons and tear size were confirmed and evaluated by high-resolution MRI with a microscopy coil. The patients were divided into three groups according to the tear size on sagittal and coronal oblique sections: small (<1 cm in maximum dimension), medium (<3 cm), and large (<5 cm). Patients with a massive tear of the rotator cuff (more than 5 cm) were also excluded from this study. Muscle atrophy was measured as the occupancy ratio of the supraspinatus, as assessed by the cross-sectional area of the supraspinatus fossa on sagittal oblique MRI (Figure 2). High-resolution MRI with a microscopy coil was used to evaluate the integrity of the intramuscular tendon of the supraspinatus. The supraspinatus tendon is known to be a five-layered structure (32). This MR technique enabled us to demonstrate a fine laminar structure and the intramuscular tendon of the supraspinatus muscle. The intramuscular tendon, which is in the supraspinatus muscle, extends to the greater tuberosity. At the tendinous region, the intramuscular tendon is continuous to the layer 2. The intramuscular tendon is recognized as a low signal intensity band (Figures 3–5).

Mann-Whitney test was used to compare the muscle atrophy of the supraspinatus, range of motion in forward elevation and external rotation, and age. All other values were compared by the chi-square test for independence. Statistical significance was set at 5% or lower. All analyses were performed using StatView software.

## Results

After conservative treatment with an average duration of 3.7 months (range, 3–10 months), 65 shoulders in 62 patients exhibited improved clinical symptoms and were classified as excellent or good. This group of patients comprised 34 males and 28 females, with an average age of 68 years (range, 45–83 years). However, 58 shoulders in 56 patients did not respond to conservative treatment and were classified as fair or poor. They eventually underwent surgical repair including arthroscopic subacromial decompression and rotator cuff repair with suture anchors. These patients were classified as the surgical group, which comprised 29 males and 27 females, with an average age of 67 years (range, 42–82 years). Supraspinatus tendon tear was identified in 39 shoulders in the conservative group and in 34 shoulders in the surgical group. Combined tear of the supraspinatus and infraspinatus tendons was observed in 26 and 24 shoulders in the conservative and surgical groups, respectively. Regarding the size of the rotator cuff tear, 20, 34, and 11 shoulders in the conservative group were classified as small, medium, and large tears, respectively, whereas in the surgical group, 21, 30, and 7 shoulders were confirmed as small, medium, and large tears, respectively (Table I). There were no significant differences between these parameters.

**Table I.** Characteristics of conservative and surgical groups.

		Conservative group	Surgical group	*P*-value
Age		68.4 years (45–83 years)	67.8 years (42–82 years)	*P* = 0.809^b^
Gender		34 males / 28 females	29 males / 27 females	*P* = 0.739^a^
Tear size	Small	20 shoulders	21 shoulders	*P* = 0.681^a^
	Medium	34 shoulders	30 shoulders	
	Large	11 shoulders	7 shoulders	
Torn tendons	SSP	39 shoulders	34 shoulders	*P* = 0.876^a^
	SSP/ISP	26 shoulders	24 shoulders	

^a^Tested by chi-square test.

^b^Tested by Mann-Whitney test.

SSP: Supraspinatus, ISP: Infraspinatus.

A history of trauma was observed in 14 shoulders (21.5%) in the conservative group, and in 14 shoulders (24.1%) in the surgical group. There were 44 shoulders (67.6%) in the conservative group that suffered from night pain, compared with 41 shoulders (70.6%) in the surgical group. The mean forward elevation angle and external rotation angle were 143 degrees and 52 degrees, respectively, in the conservative group, and 135 degrees and 35 degrees, respectively, in the surgical group at the time of initial consultation. Impingement sign was positive in 20 shoulders (30.7%) in the conservative group, compared with 46 shoulders (79.3%) in the surgical group.

Regarding muscle atrophy of the supraspinatus, the occupancy ratio of the supraspinatus, as assessed by the cross-sectional area of the supraspinatus fossa on sagittal oblique MRI, was 78.0% (range 50%–95%) and 69.8% (range 30%–91%) in the conservative and surgical groups, respectively. Intact intramuscular tendon of the supraspinatus was observed in 38 shoulders (58.4%) in the conservative group, whereas 14 shoulders (24.1%) in the surgical group exhibited an intact intramuscular tendon. There were significant differences between the groups for the external rotation angle, impingement sign, muscle atrophy of the supraspinatus, and integrity of the intramuscular tendon of the supraspinatus (Table II).

**Table II.** Statistical analysis of factors related to successful outcome of conservative treatment.

	Conservative group	Surgical group	*P*-value
History of trauma	21.5% (14/65 shoulders)	24.1% (14/58 shoulders)	*P* = 0.731^a^
Night pain	67.6% (44/65 shoulders)	70.6% (41/58 shoulders)	*P* = 0.719^a^
Range of motion in forward elevation	143.4 ± 25.5 (50–180)	135.3 ± 25.9 (80–175)	*P* = 0.077^b^
Range of motion in external rotation	52.2 ± 14.6 (15–80)	35.0 ± 20.1 (0–80)	*P* < 0.001^b^
Impingement sign	30.7% (20/65 shoulders)	79.3% (46/58 shoulders)	*P* < 0.001^a^
Muscle atrophy	78.0 ± 10.8% (50%–95%)	69.8 ± 14.7% (30%–91%)	*P* = 0.031^b^
Intramuscular tendon	58.4% (38/65 shoulders)	24.1% (14/58 shoulders)	*P* < 0.001^a^

^a^Tested by chi-square test.

^b^Tested by Mann-Whitney test.

## Discussion

Much controversy exists as to the management of full-thickness tears of the rotator cuff. Not all patients with rotator cuff tears require surgical treatment. However, there have been fewer reports about conservative treatment for rotator cuff tears, despite the prevalence of studies regarding surgical treatment. Therefore, further research is necessary regarding the effectiveness of conservative treatment.

According to the previous reports, the success rates of conservative treatment can vary widely. Brown (17) reported that 87% of patients with rotator cuff injury who had full range of abduction at initial examination regained full function by conservative treatment and concluded that the total success rate of conservative treatment was 70%. Itoi and Tabata (20) also reported a high percentage (82%) of recovery after conservative treatment. Samilson and Binder (24) concluded that 59% out of 194 shoulders obtained good results, whereas Wolfgang (13) reported the recovery rate of patients to be 33%. The success rate for conservative treatment in our series was 65 out of 123 shoulders (52.8%). This success rate is not as high as those of previous studies, likely due to differences in the methods utilized for conservative treatment, such as differences in physical therapy (17,20,23) and in the indication for conservative and surgical treatment. Another reason for these results may be associated with our inclusion/exclusion criteria. The cases of partial-thickness tears were not included in our study.

Some authors have described the factors related to successful outcome for conservative treatment for full-thickness rotator cuff tears. Itoi and Tabata (20) demonstrated that patients who responded well to conservative treatment exhibited a good range of motion and strength of abduction at initial examination. Minagawa and colleagues (23) reported that restricted range of motion in external rotation and tear extension from the supraspinatus to the infraspinatus tendon negatively affected the outcome following conservative treatment.

In this study, we evaluated the intramuscular tendon of the supraspinatus by high-resolution MRI with a microscopy coil. The rotator cuff is known to be a five-layered structure (32). The second layer is composed of clearly packed, parallel tendon fibers grouped in large bundles. These bundles extend directly from the supraspinatus, infraspinatus, and subscapularis muscle belly to the greater tuberosity and lesser tuberosity (Figure 3). The patients with normal integrity of intramuscular tendon responded well to the functional exercises of scapula and inner muscles. Intramuscular tendons of the supraspinatus, infraspinatus, and subscapularis have been worthy of note as functional structures, which centralize the humeral head in the glenoid cavity. Our study revealed that the integrity of the intramuscular tendon correlated with a successful outcome following conservative treatment. This result suggests that the intramuscular tendon plays an important role for functional structure of the shoulder. Our observation is the first to demonstrate that the integrity of the intramuscular tendon of the supraspinatus affects the outcome of conservative treatment.

In this study, the following four factors correlated very well with a successful outcome following conservative treatment: 1) a preserved range of motion in external rotation (more than 52 degrees); 2) negative impingement signs; 3) little or no atrophy of the supraspinatus muscle (the occupancy ratio of the supraspinatus more than 78%); and 4) preserved intramuscular tendon of the supraspinatus (Table III). Patients with at least three of these four factors were observed in 33 shoulders in the conservative group and in 5 shoulders in the surgical group. The success rate of conservative treatment was 87% in the cases with at least three of these four factors. Preserved range of motion in external rotation indicates little or no capsular contracture and stiffness of the subscapularis muscle, and good function of the infraspinatus and teres minor muscles. These muscles seem to keep the humeral head well centered in the glenoid cavity and prevent impingement between the humeral head and the acromion. Intact intramuscular tendon of the supraspinatus may indicate the potential to respond well to conservative treatment.

**Table III.** Statistical analysis of odds ratios of four factors.

	Odds ratio	*P*-value
Impingement sign	8.62	*P* < 0.001
Intramuscular tendon	4.42	*P* < 0.001
Range of motion in external rotation	3.69	*P* < 0.001
Muscle atrophy	2.28	*P* = 0.024

On the other hand, a history of trauma and night pain did not correlate with the outcome of conservative treatment. Previous reports of acute rotator cuff tears suggested that earlier surgical intervention resulted in good outcome (1). However, acute rotator cuff tears associated with the previous four factors responded to conservative treatment in this series. Therefore, in patients with these four factors, conservative treatment should be implemented as the first choice regardless of the presence of a history of trauma or night pain.

There were several limitations in the present study. The first one is that we did not evaluate the function of the scapula although the scapular motion is known to be important to support rotator cuff function. A second limitation is that we identified the intramuscular tendon of only the supraspinatus. All the cuff muscles have intramuscular tendons. As mentioned previously, the subscapularis and infraspinatus muscles play an important role in stabilizing the glenohumeral joint. The association between the integrity of other intramuscular tendons and the clinical outcome of rotator cuff tears needs to be investigated in the future.

In summary, among patients with full-thickness tears of the rotator cuff, those with intact intramuscular tendon of the supraspinatus, little or no atrophy of the supraspinatus muscle, negative impingement signs, and preserved motion in external rotation are more likely to have a successful outcome following conservative treatment than those without these four factors. These four factors are useful in selecting patients who will respond well to conservative treatment before initiating the treatment.
